# Hollow Complete Denture With a Speech Bulb Prosthesis: A Case Report

**DOI:** 10.7759/cureus.55671

**Published:** 2024-03-06

**Authors:** Shubha D Sarmalkar, Meena Aras, Aradhana Nagarsekar, Praveen Rajagopal

**Affiliations:** 1 Prosthodontics, Goa Dental College and Hospital, Goa, IND

**Keywords:** lightweight prosthesis, cleft palate, speech therapy, palatal obturator, velopharyngeal insufficiency

## Abstract

Speech is the most basic yet invaluable mode of expression for an individual. Alterations in speech can have vast effects on the psychological well-being of a person, hampering social interactions. Congenital or traumatic defects of the hard and soft palate result in velopharyngeal dysfunction, which often results in abnormal and aberrant speech. Apart from these, it is also a common outcome following surgical repair of cleft palate. Prosthodontic management of such cases with velopharyngeal obturators to improve speech and function is well documented and known to give optimal results. In this case report, we are presenting the rehabilitation of residual velopharyngeal insufficiency post-cleft palate closure using a speech bulb prosthesis attached to a complete denture. As the speech bulb would add to the weight of the existing prosthesis, a hollow complete denture was planned. The prosthesis resulted in a decrease in nasal air emissions and hypernasality, thus improving the patient’s communication skills and overall quality of life.

## Introduction

Velopharyngeal dysfunction is a collective term used to describe a group of disorders that result in the leakage of air into the nasal passage during speech [1]. Along with lateral and posterior pharyngeal walls, the soft palate forms a muscular valve termed velopharyngeal sphincter (VPS) which divides the pharynx into oral and nasal parts [2]. Normal functioning of this sphincter aids in speech apart from the prevention of nasal regurgitation of food and liquids. As a result of abnormal functioning of VPS, speech samples can demonstrate hypernasality, excessive nasal air emissions, articulation defects, and poor intelligibility. These variations from normal speech have immense effects on the psychological well-being of an individual, adding to the existing physical disability [3]. Palatal defects can be classified either as congenital (cleft palate) or acquired (resulting from surgeries involving parts of the palate). Structural/anatomical defects of the soft palate result in velopharyngeal insufficiency, whereas velopharyngeal incompetency is a result of a neurological deficit [4,5]. Cleft palate patients often present with residual velopharyngeal inadequacy following surgical repair [3].

## Case presentation

**Figure 1:**
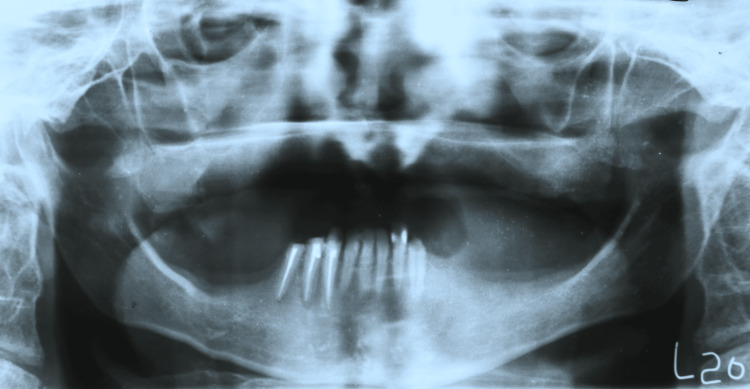
Orthopantomagram.

**Figure 2:**
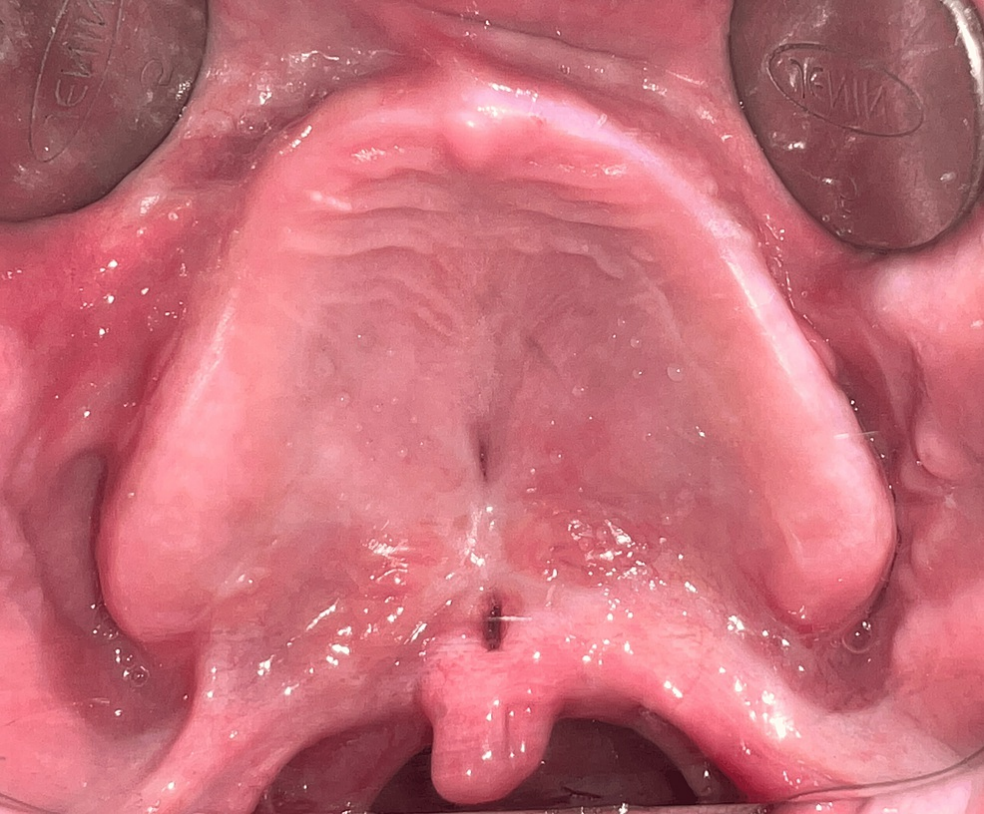
Maxillary edentulous arch.

**Figure 3:**
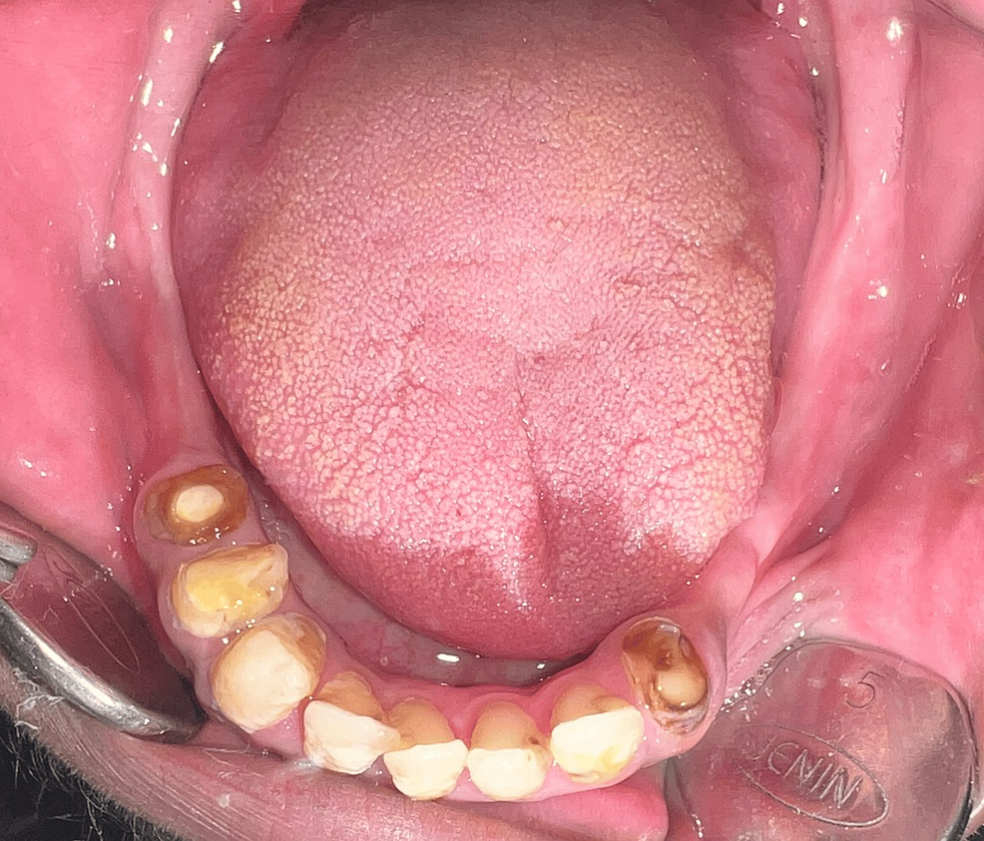
The mandibular arch.

A 35-year-old male diagnosed with achondroplasia, presenting with a short stature, reported to us with a chief complaint of unretentive upper denture and unintelligible speech. On examination, the maxillary arch was completely edentulous, and the mandibular arch was partially edentulous (Kennedy’s class one) (Figures 1-3).

On eliciting history, it was found that the patient had undergone a cleft palate (Veau’s class one) repair surgery three years back. The patient had undergone cleft palate closure in childhood, but relapse of the defect demanded a second repair surgery. The complete denture was fabricated before the second surgical closure and was not retentive at the time of presentation. The patient had unintelligible speech caused by poor articulation and hypernasality and expressed interest in correction of the same. A detailed analysis led to a diagnosis of palatopharyngeal insufficiency. The patient was explained about surgical closure as well as prosthetic rehabilitation options, including maxillary semi-fixed (overdenture) and removable complete denture along with a speech bulb. Although a treatment plan consisting of maxillary implant-supported overdenture with a speech bulb and a lower cast partial denture would be considered ideal in such cases, considering the patient’s history of eventful previous extractions and desaturation in the operation theater under general anesthesia, any treatment plan involving surgery was ruled out. A maxillary removable complete denture with a speech bulb and a mandibular removable partial denture was planned. As the speech bulb would add to the weight of the prosthesis compromising its retention further, a hollow complete denture with a speech bulb was considered.

**Figure 4:**
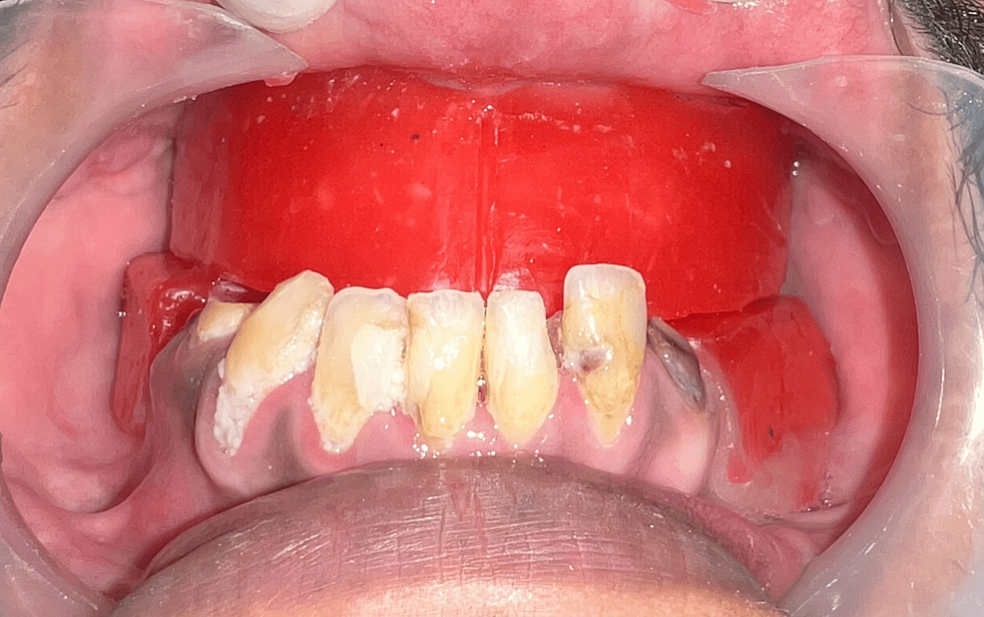
Jaw relation record.

**Figure 5:**
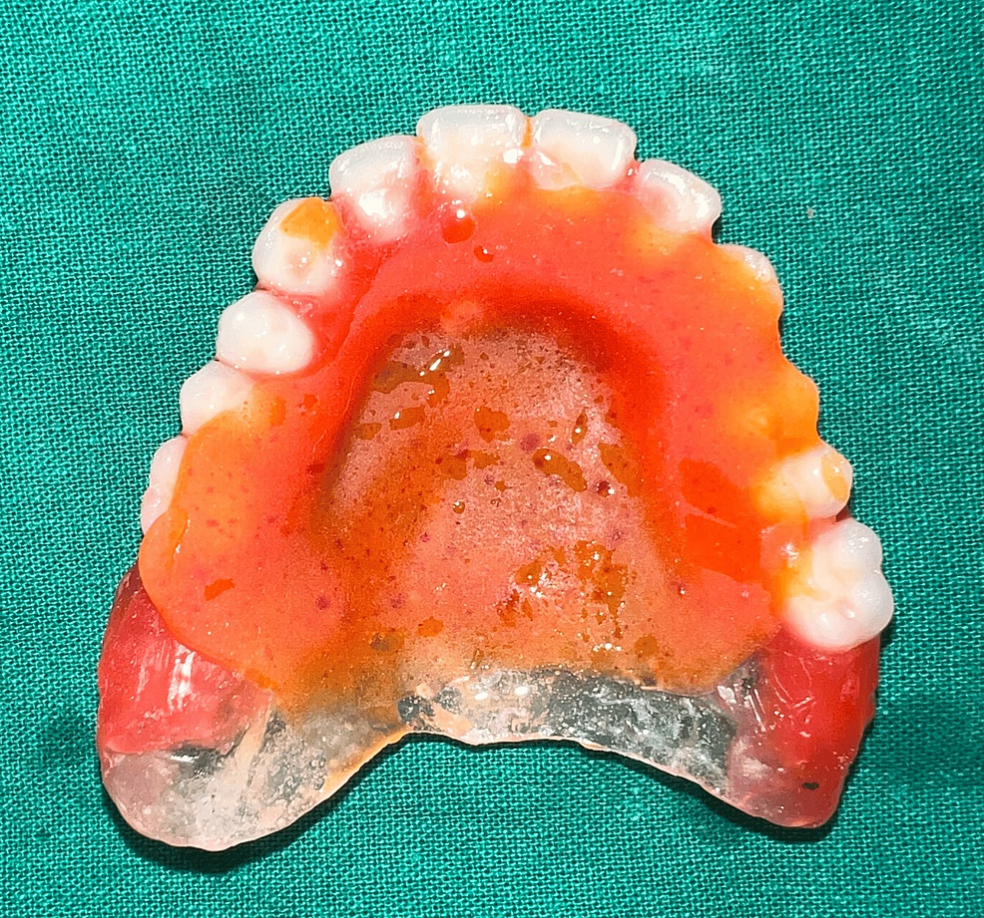
Palatogram assessment.

Following primary impression of the maxillary arch with impression compound ( Y-Dents, MDM) and mandibular arch with alginate (Tropicalgin, Zhermack), border molding and secondary impressions were performed using low fusing impression compound (DPI pinnacle) and PVS light body (elite HD+, Zhermack), respectively, in a conventional manner. Jaw relation was recorded at the desired VD and try-in was done (Figure 4). Following this, a palatogram assessment was performed using tissue conditioner (visco-gel, Dentsply) and edible food coloring to check if any refinement of palatal contours was needed [6] (Figure 5). The patient was engaged in a conversation for a few minutes with the tissue conditioner in the palatal area and the displacement of the same at the end of the session was assessed. No significant displacement of the material was noted.

**Figure 6:**
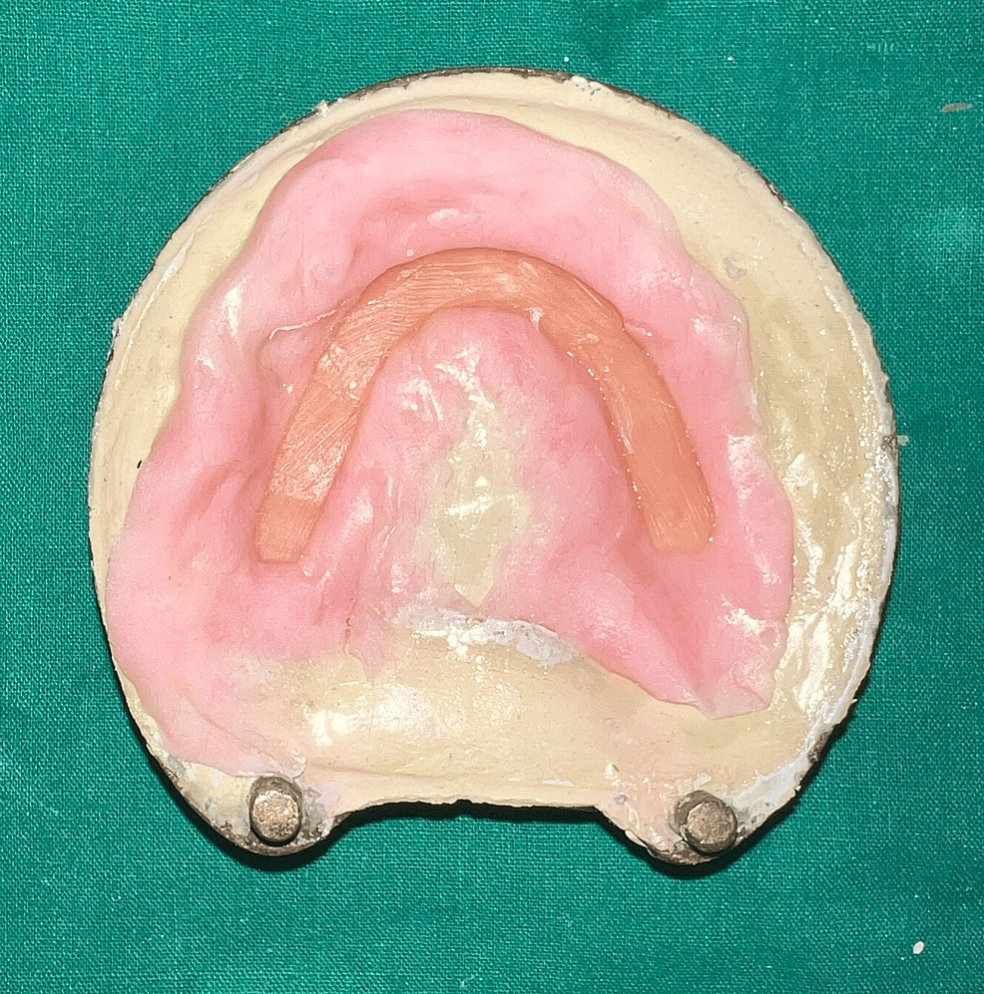
Glycerin soap spacer during the packing stage.

**Figure 7:**
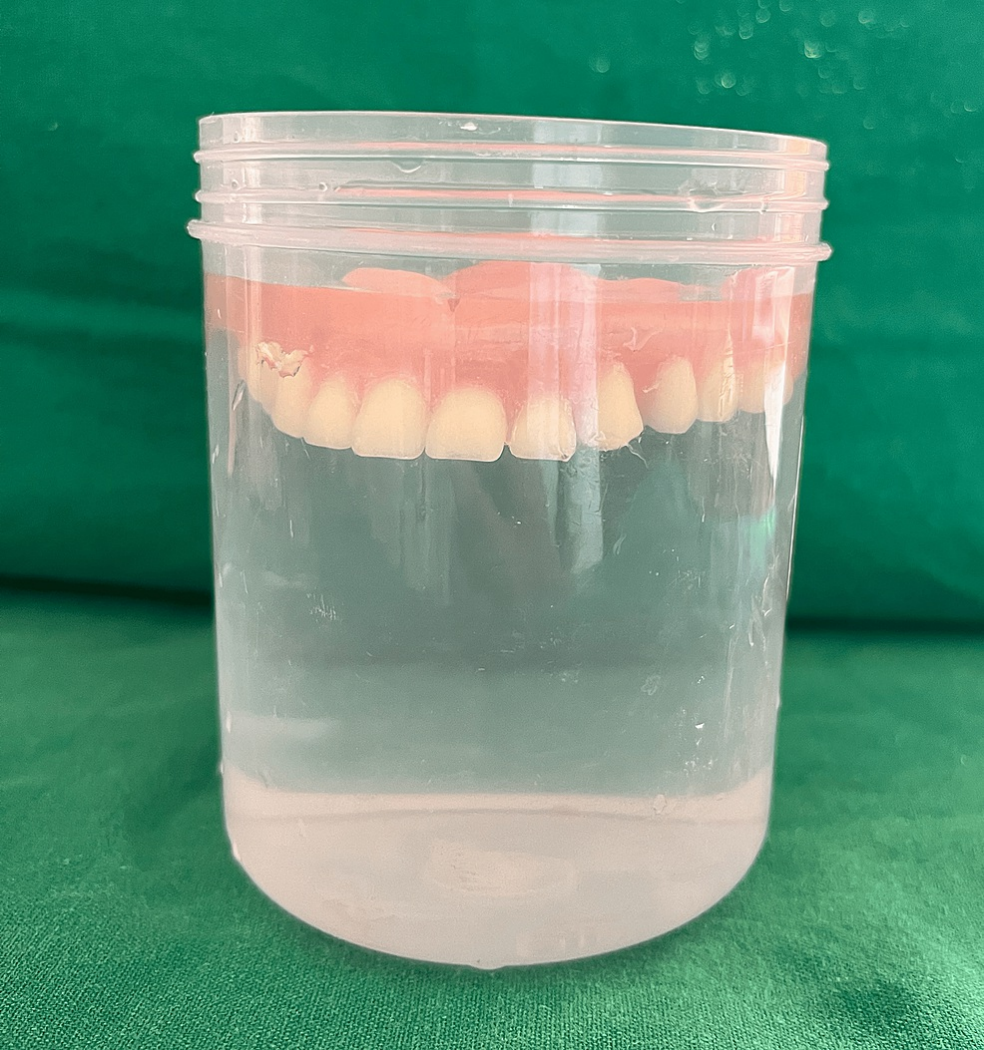
Confirmation of denture hollowness.

To reduce the weight of the final prosthesis, a hollow complete denture was planned. Various materials have been used in the literature previously for the same, including salt, putty, caramel, and asbestos. Glycerin soap was preferred due to ease of placement and removal while maintaining the desired volume. The glycerin soap was carved in the desired shape and thickness keeping in mind the denture dimensions and packed along with heat cure acrylic such that there would be no exposure of the same. Later, the same was retrieved using hot water and the opening was sealed. Hollowness of the denture (empty volume within the denture) was confirmed by immersing it in a container of water where no air bubbles were noted [7] (Figures 6, 7).

**Figure 8:**
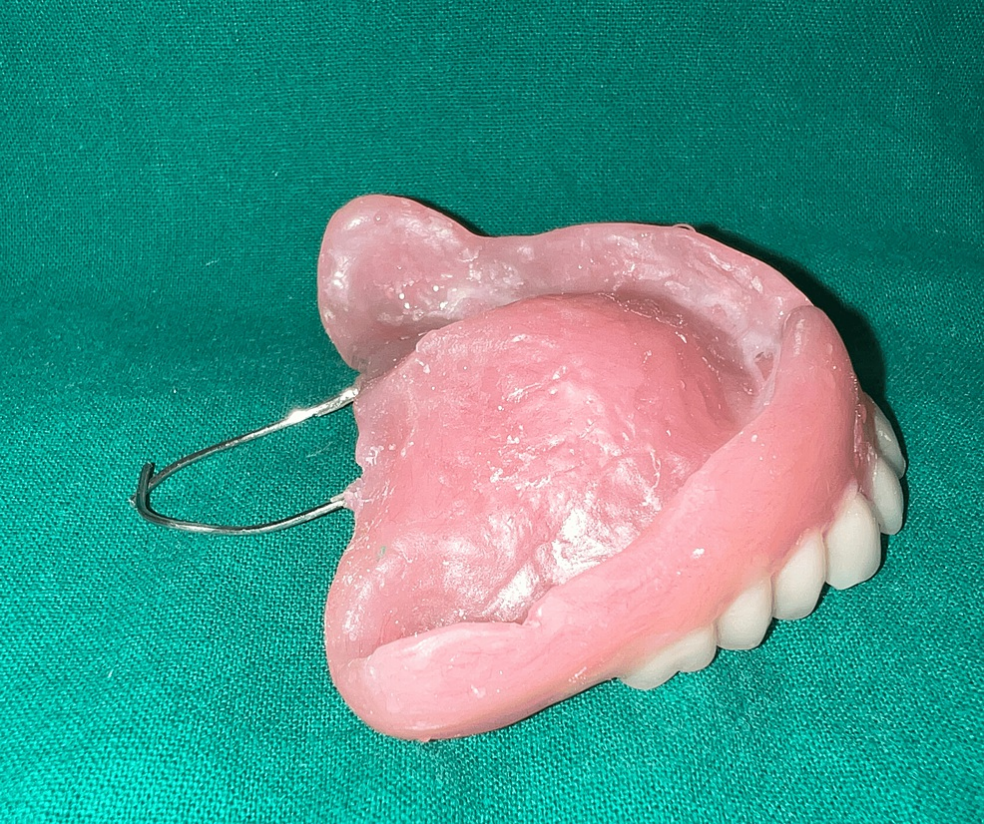
Denture with the addition of wire loops to support low fusing impression compound.

The dentures were delivered and the patient was recalled after a week for the fabrication of the speech bulb. On recall appointment, a 20-gauge stainless steel wire in the form of a loop was attached to the posterior end of the denture to hold the low fusing compound (DPI pinnacle) in position during functional molding of the defect area [8] (Figure 8).

**Figure 9:**
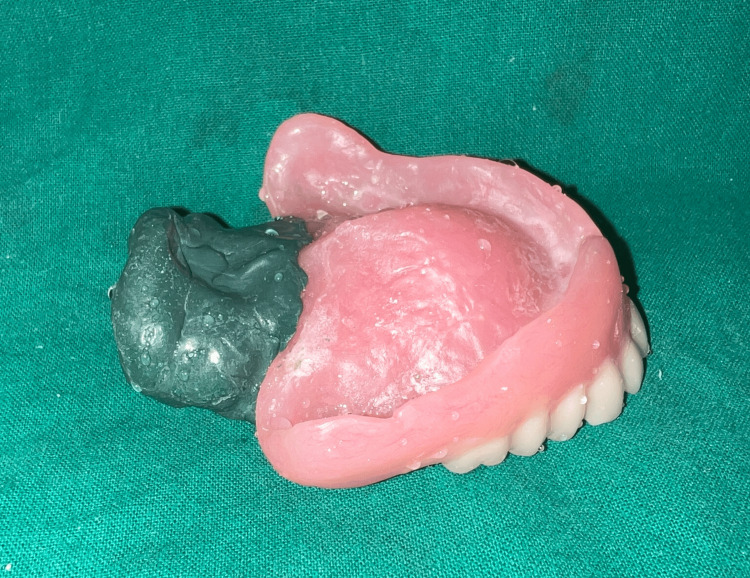
Impression of the velopharyngeal defect.

The patient was asked to move his head side to side in a circular manner extending it as far forward and backward as possible. The patient was also instructed to say “ah” and swallow [8] (Figure 9). The impression compound was preferred due to its high viscosity and reduced flow avoiding backflow of the impression material.

**Figure 10:**
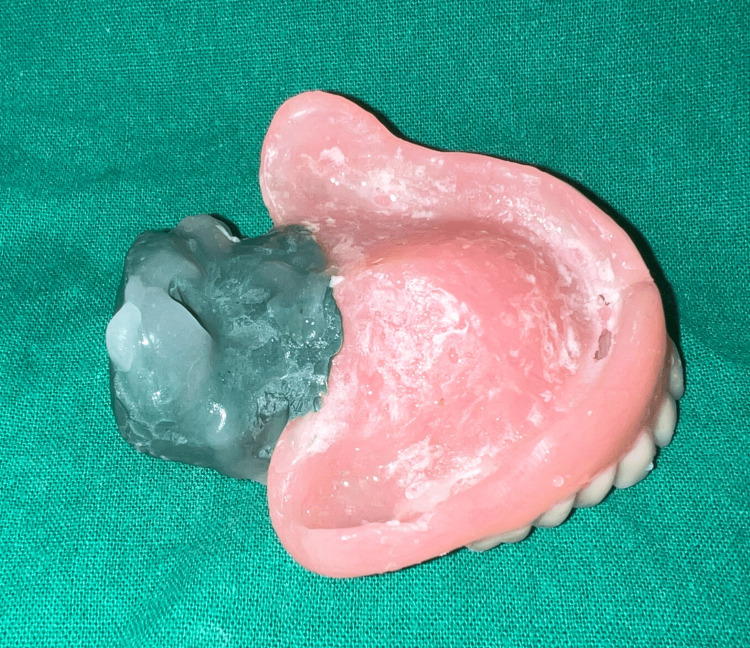
Impression of the velopharyngeal defect relined with a tissue conditioner.

**Figure 11:**
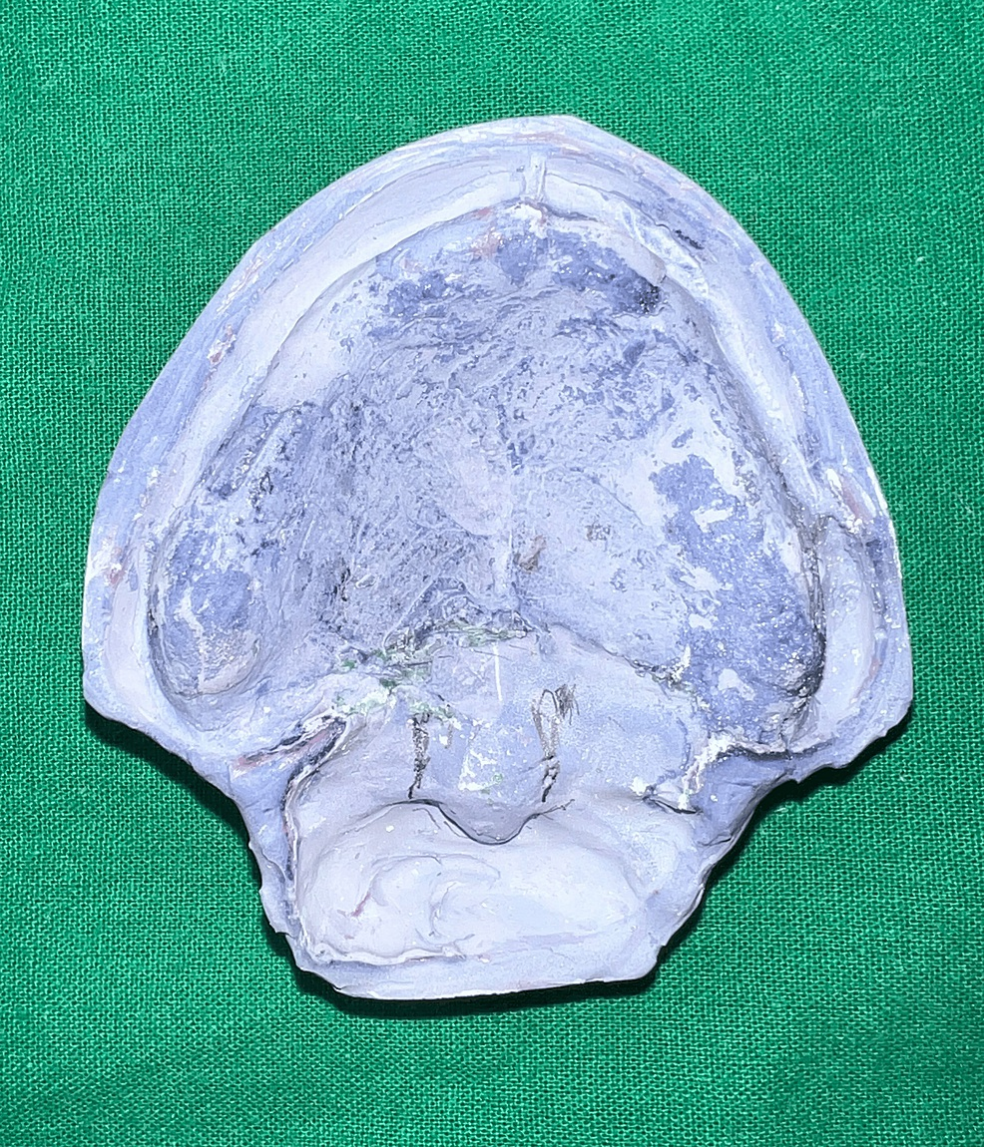
Stone cast.

Functional impression was further relined using tissue conditioner (visco-gel, Dentsply) while repeating the above-mentioned movements [9] (Figure 10). Following beading and boxing, the impression was poured using type three dental stone (Goldstone) (Figure 11).

**Figure 12:**
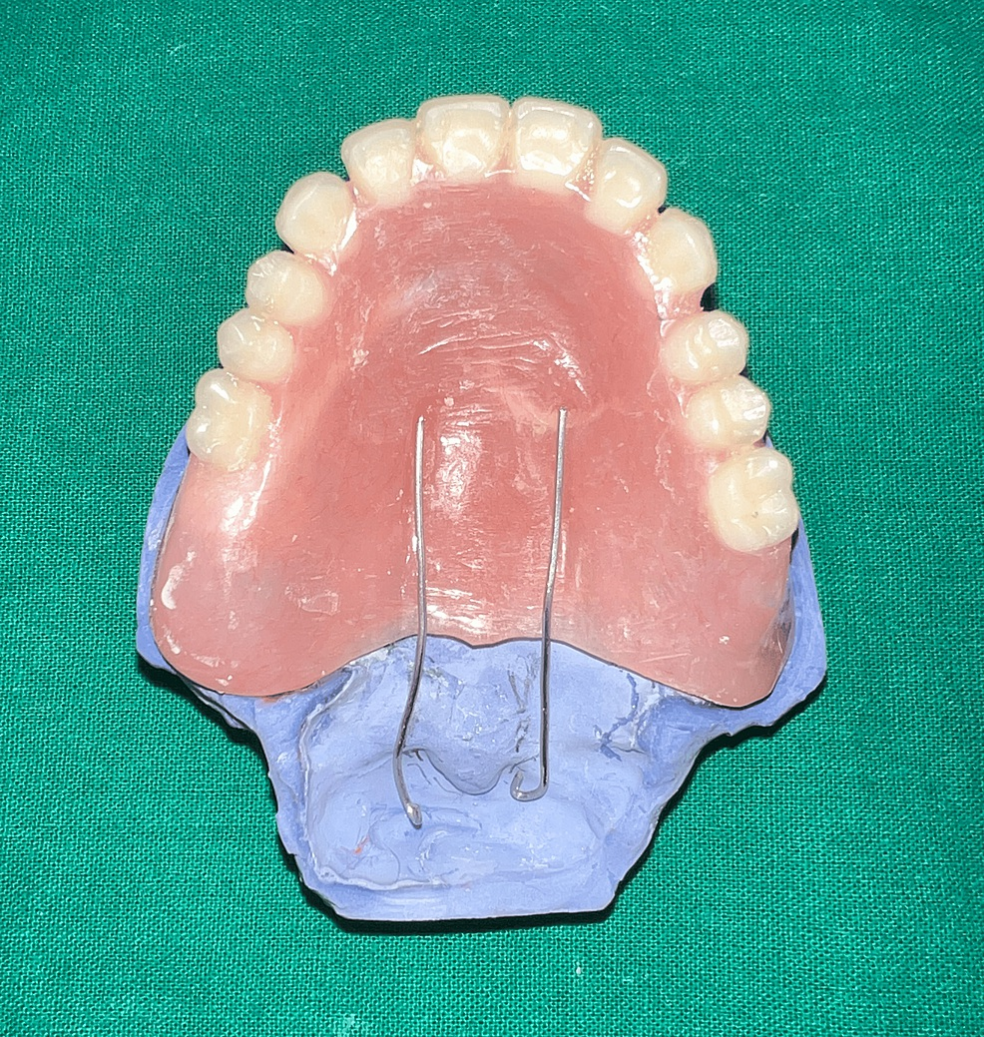
Relieving the uvula in the final prosthesis using two wire extensions from the denture.

As the uvula of the patient was prominent and coverage of the same was anticipated to further compromise retention of the prosthesis, we decided on relieving it in the final prosthesis. Thus, two 1 mm stainless steel wire loops were arranged connecting the denture with the obturator bulb while relieving the uvula (Figure 12).

**Figure 13:**
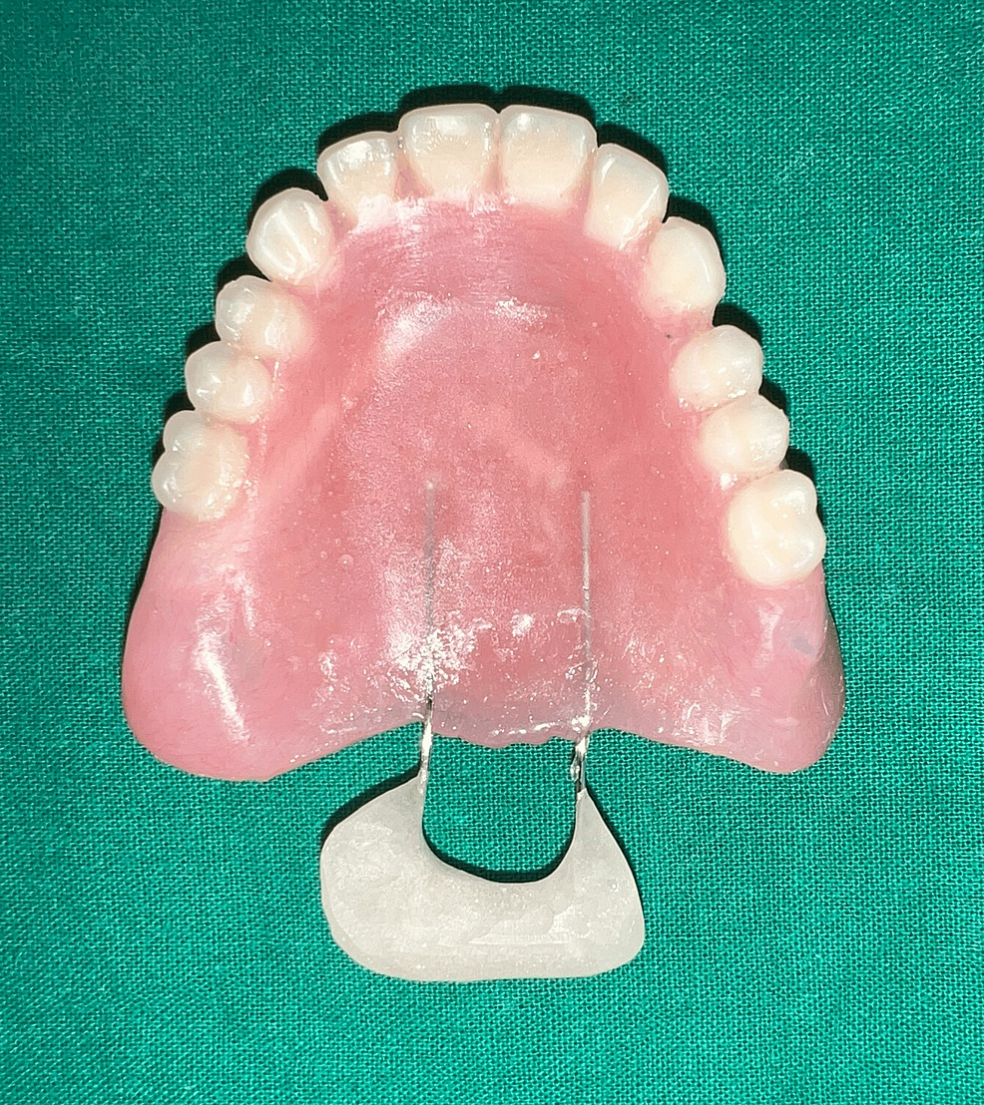
Final prosthesis: hollow complete denture with speech bulb.

**Figure 14:**
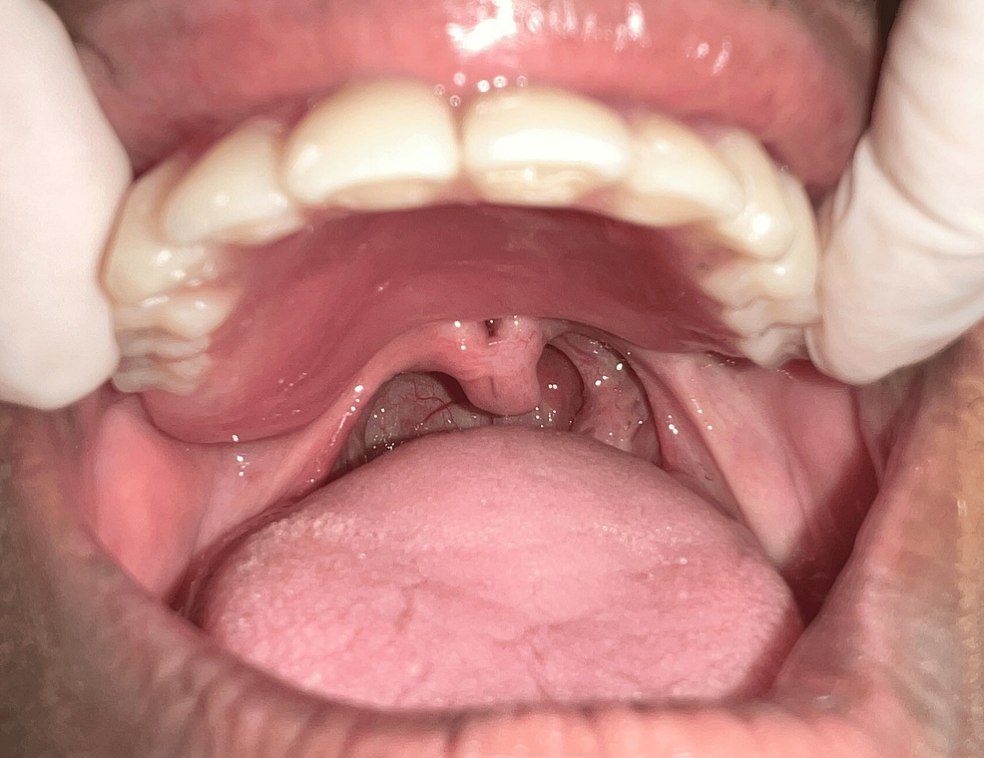
Velopharyngeal defect without speech bulb prosthesis.

The speech bulb was fabricated with cold cure acrylic and checked in the patient’s mouth for any discomfort or pain, and following the adjustment, it was delivered to the patient (Figure 12). The defect and closure of the same can be appreciated in Figure 13 and Figure 14. The other option included the fabrication of the speech bulb in heat cure acrylic. In this case, cold cure was preferred due to ease of fabrication considering the additional wire component of the prosthesis and owing to the small size of the defect. The final prosthesis in occlusion can be seen in Figures 16-18.

**Figure 15:**
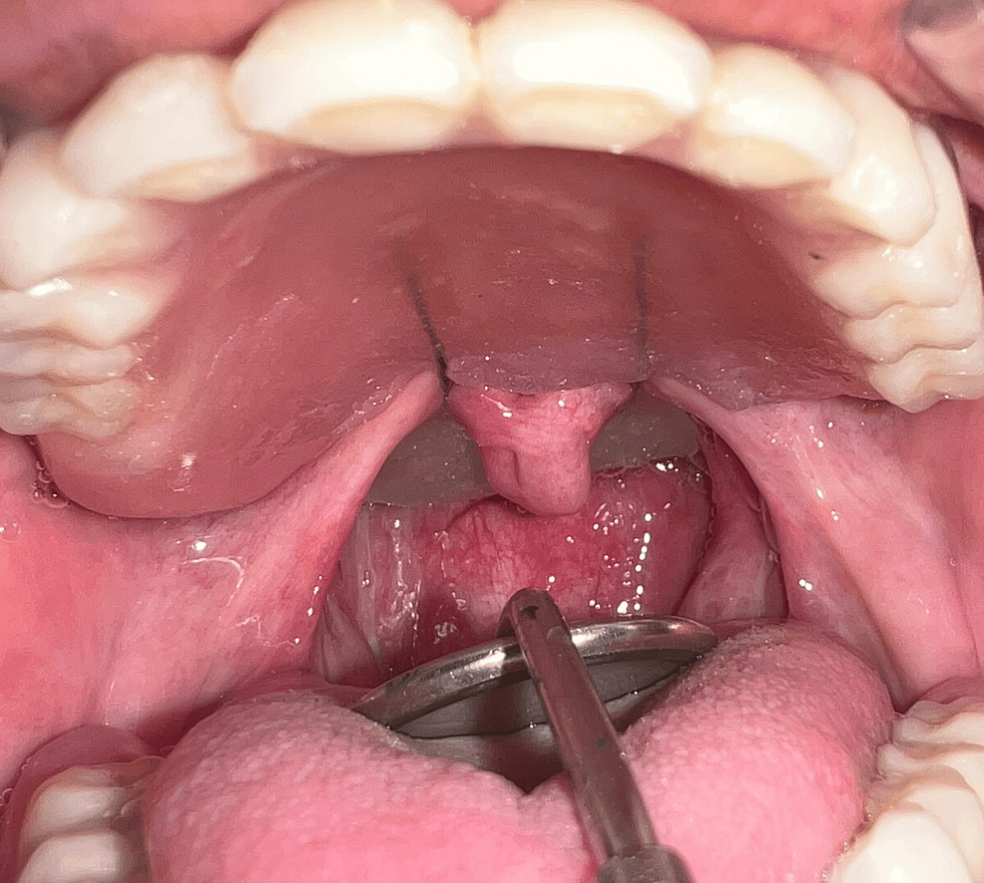
Velopharyngeal defect closure with speech bulb prosthesis.

**Figure 16:**
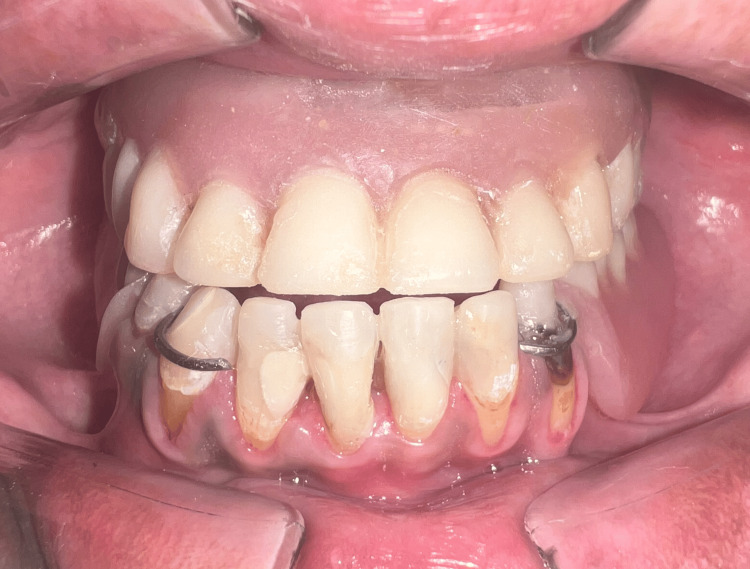
Upper complete denture and lower removable partial denture in occlusion: frontal view.

**Figure 17:**
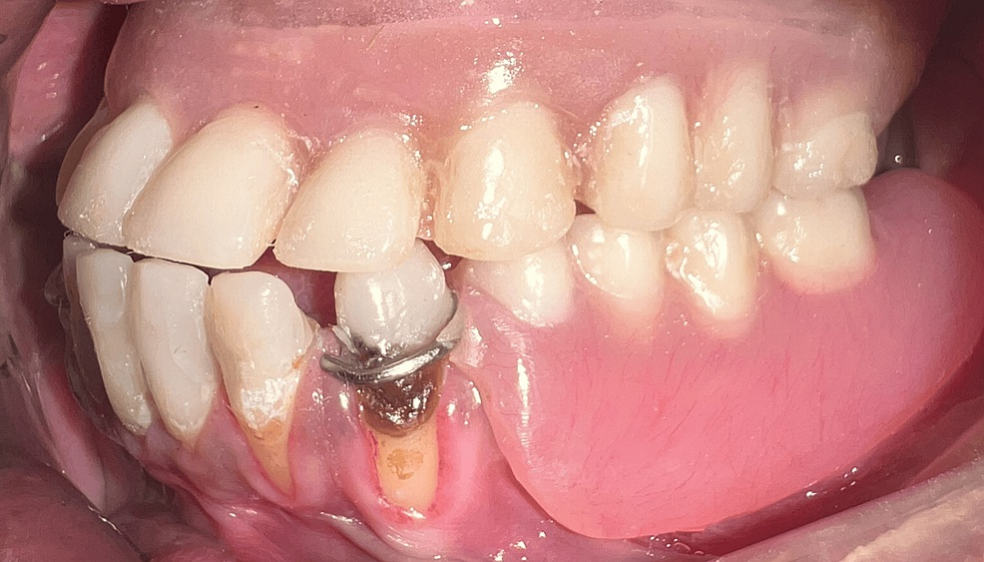
Upper complete denture and lower removable partial denture in occlusion: left lateral view.

**Figure 18:**
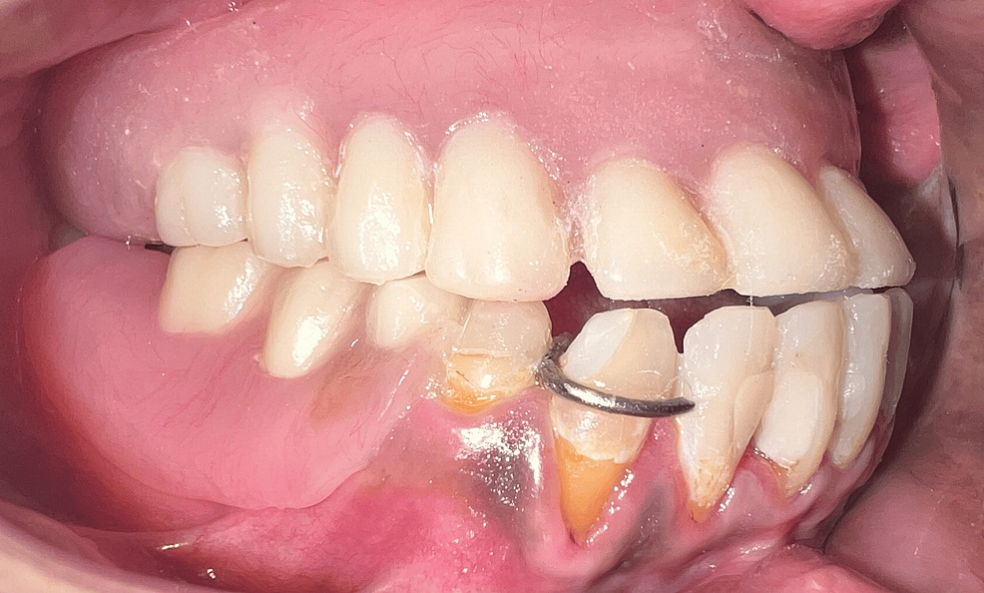
Upper complete denture and lower removable partial denture in occlusion: right lateral view.

The patient was analyzed by a speech therapist on four occasions, namely, the first visit before initiation of treatment, post-delivery of the complete denture, following the addition of speech bulb prosthesis, at one-week follow-up. The next follow-up with the speech analyst is expected at the end of six months. The prosthesis led to improvement in speech and a reduction in nasality and nasal air emissions, as analyzed by the speech therapist. Further, the speech intelligibility also improved (as reported by family members) and the patient was happy with the outcome of the prosthesis. The speech evaluation criteria and speech intelligibility by the Ali Yavar Jung National Institute of Speech and Hearing Disabilities (AYJNISHD) rating scale are presented below (Table 1) [10].

**Table 1:** Speech intelligibility: Ali Yavar Jung National Institute of Speech and Hearing Disabilities rating scale (2003).

Score	Interpretation
0	Normal
1	Can understand with little effort; however, speech is not normal
2	Can understand with little effort, but occasionally need to ask for repetition
3	Can understand with concentration and effort, especially by sympathetic listeners
4	Can understand with difficulty and concentration by family but not others
5	Can understand with effort if the content is known
6	Cannot understand at all even if the content is known

Speech evaluation

Subjective assessment of following parameters was performed by a single speech therapist: (1) Articulation: photo-articulation test (evaluation of substitutions, omissions, distortions, and additions); (2) nasality: auditory-perceptual evaluation of voice (Cape V); (3) nasal air emissions: assessment of nasal air emission after pronunciation of explosive bilabials (ph, bh); (4) speech intelligibility: the AYJNISHD rating scale (2003).

The patient’s speech was found to have distortions and omissions. However, the nasality and nasal air emissions were reduced from severe to moderate following the placement of the obturator. The AYJNISHD rating for speech intelligibility went from score 5 to score 3 which was a noticeable change. Apart from these improvements, the patient also mentioned a significant reduction in nasal regurgitation of liquids and semisolids following delivery of the prosthesis leading to a better quality of life.

## Discussion

Velopharyngeal assessment comprises perceptual speech evaluation and functional imaging which includes video nasoendoscopy, speech videofluoroscopy, multiview videofluoroscopy, and nasoendoscopy. These are used as diagnostic aids for confirming impaired velopharyngeal function before decision-making regarding the treatment plan [1]. The velopharyngeal defects can either be corrected surgically or managed prosthetically. Pharyngeal flap, sphincter pharyngoplasty, posterior pharyngeal wall augmentation, and Furlow double-opposing Z-plasty are various surgical options mentioned by Yamaguchi et al. [11]. Prosthetic management of a velopharyngeal defect is a predictable treatment option if surgical options are either refused by the patient or are contraindicated due to reasons such as compromised oral and systemic health, massive size of the defect, etc. [9]. In the present case, surgical options were not considered due to the patient’s altered and eventful response to previous surgeries. Prosthetic options to restore defects of the hard and soft palate include speech bulbs or velopharyngeal obturators. Objectives of a velopharyngeal obturator are to provide control of the nasal air emissions, reduce inappropriate nasal resonance during speech, and prevent the leakage of food and liquids into the nasal passage during deglutition [8]. Partially edentulous cases rehabilitated with velopharyngeal obturators make use of cast partial denture components for added retention [5]. Few completely edentulous cases that have been rehabilitated in the literature have used conventional complete denture prostheses along with speech bulbs [5,8,12]. One of the cases also mentions the fabrication of a hollow speech bulb to further reduce the weight of the final denture [13]. Achieving effective retention by conventional prostheses for edentulous arches with both hard and soft palate defects is often very difficult. As the discussed case did not have a hard tissue defect, retention was predictable to some extent. However, considering the ridge form and anatomy of the hard palate which precluded achieving good retention, it was improved by reducing the weight of the prosthesis by fabrication of a hollow complete denture. In this case, a low-fusing impression compound was used for functional contouring of the palatal defect, which was further relined with tissue conditioner. Impression waxes, zinc oxide eugenol impression paste, monophase, vinyl polysiloxane, or polyether impression materials may also be used to make the final impression [1,3]. In the described case, the patient was asked to drink water post-prosthesis insertion to test the complete closure of the defect. No water reflux into the nasal cavity was seen. Participation of a speech pathologist in the treatment of these cases to test articulation errors and inappropriate oronasal resonance balance is a must for a better prognosis [14]. Speech therapy post-rehabilitation is necessary for continued improvements. Apart from these, a few exercises including auditory feedback and self-assessment, pinching of the nose during sound production, and comparing it with routine speech are advised. Although we achieved success by a significant margin in this case, the results are not always predictable. Almost one-third of the patients rehabilitated for reduction of hypernasality and speech improvements do not show positive results [15,16]. Several factors either individual or in combination including persistent compensatory articulation, presence of severe dysphonia, and failure of the speech bulb to adequately close the velopharyngeal gap, could result in failures [16].

## Conclusions

In this report, the velopharyngeal insufficiency was successfully rehabilitated by a hollow complete denture with a speech bulb prosthesis. It led to a significant decrease in hypernasality and nasal air emissions and the patient was happy with the outcome. As the patient underwent closure of the cleft palate at a later age, the projected improvements in speech are expected to take longer. However, during the post-insertion period, improvements were noted, though the continued intervention of a speech therapist will be needed. Apart from speech therapy sessions, the patient was also advised to continue with the mentioned exercises. The patient also mentioned a significant reduction in nasal regurgitation of liquids and semisolids following prosthesis delivery improving his quality of life. Limitations of this case report include the fabrication of a speech bulb with a cold cure, which might require close follow-ups to check for any changes due to leaching out of the monomer. Moreover, as the patient presented with economic constraints, a lower cast partial denture could not be fabricated, which could be considered in the future.
